# Excess all-cause mortality in Norway in 2024

**DOI:** 10.1177/14034948251371830

**Published:** 2025-09-08

**Authors:** Richard Aubrey White, Anders B. Nygaard, Arne Søraas, Gunhild A. Nyborg

**Affiliations:** 1 Independent Researcher, Oslo, Norway; 2 Department of Microbiology, Oslo University Hospital, Oslo, Norway

**Keywords:** Excess mortality, COVID-19, all-cause mortality, long COVID, pandemic policy, post-acute sequelae, post-acute sequelae of COVID-19

## Abstract

**Aims:**

The Norwegian Institute of Public Health calculated excess mortality for Norway in 2024 using a reference period that included 2023—a year with significant excess mortality—and concluded there was no excess mortality in 2024. This study estimates excess mortality in 2024 using only pre-pandemic years as the reference, providing a basis for identifying excess COVID-19 related mortality.

**Methods:**

We estimated excess mortality in 2024 using a negative binomial model trained on 2010–2019 data. Deaths were modelled by age (0, 1–19, 20–39, 40–64, 65–79, 80–89 and 90+ years) and sex, with population offsets. Expected mortality was projected using both a conservative approach where the prediction for 2023 was carried forward to 2024 and a non-conservative linear extrapolation to 2024.

**Results:**

The conservative approach estimated 2898 excess deaths (7.0%; 95% prediction interval (PI), 4.9–9.1%) in 2024. Significant excess mortality was observed in age groups 1–19 (45 deaths; 36.6% excess), 20–39 (107 deaths; 17.6% excess), 40–64 (439 deaths; 10.6% excess) and 65–79 (1631 deaths; 13.7% excess). Ages 1–39 and 40–64 accounted for approximately 5% and 15% of total excess mortality, respectively.

**Conclusions:**

**Persistent excess mortality from 2022 to 2024 suggests a new elevated mortality baseline and a reduction or reversal of Norway’s pre-pandemic mortality decline. Although multiple factors may contribute, given sustained excess mortality since 2022, our findings suggest that the unmitigated spread of SARS-CoV-2 in Norway since 2022 can be associated with increased mortality, particularly for those under 65**.

## Background

Excess mortality occurs when deaths exceed expected levels based on historical trends. Expected death trends typically reflect declining mortality rates in most ages, although total death counts may increase due to both population aging and an overall growing population. Excess mortality is a key indicator of pandemic impact, including both direct and indirect deaths, such as from disrupted healthcare, long-term post-infection effects or societal changes.

Long-term mortality trends in Norway (and in most countries) show consistent declines across most age groups over recent decades [1], although patterns vary by age, with less improvement among younger populations, where external causes of death are more prominent.

In 2020–2021, Norway implemented effective infection control measures against COVID-19. No excess mortality was observed during this period [2]. In early 2022, the authorities changed strategy, considering frequent SARS-CoV-2 infections desirable to maintain herd immunity to protect the healthcare system against major COVID-19 waves [3–5]. This strategy differs notably from World Health Organisation and other international guidelines [6].

SARS-CoV-2 infection causes long-term effects influencing morbidity and mortality on a large scale [7]. Even mild-to-moderate COVID-19 increases mortality risk for up to one year after infection [8]. Vaccination reduces this risk [8–10], including in children [11,12].

Excess mortality in 2022 and 2023 was observed at 11.5% and 5.6%, respectively [2,13]. In 2023, 22.4% more deaths from cardiovascular disease than expected were observed across all ages [14]. Cardiovascular disease is a well-known COVID-19 sequela [8]. Additionally, 36% excess mortality was observed in 1–39-year-olds, explained partially by 51.2% more deaths from disease than expected in this age group [14].

The Norwegian Institute of Public Health’s (NIPH) risk assessment of Norway’s COVID-19 strategy excluded long-term consequences of COVID-19, focusing only on hospitalisations, intensive care capacity and acute deaths [4]. When NIPH presented estimates for excess mortality in 2024, they included 2023 in the reference period [15]. However, in 2023 there were high levels of SARS-CoV-2 spread and significant excess mortality. Including 2023 in the reference year results in a higher baseline, and thus lower estimated excess mortality. NIPH’s method is therefore suitable only for detecting acute changes in mortality. This risks incorrect conclusions if such a method is used to evaluate the current COVID-19 strategy. Nevertheless, NIPH’s conclusion was: ‘in 2023 there was . . . an excess mortality in the age group under 40 years . . . in 2024 NIPH finds no excess mortality for any specific age groups, not even those under 40 years’ [16]. A more accurate interpretation of NIPH’s results is rather that mortality in 2024 was not significantly higher than in 2023. This may suggest a new, elevated mortality baseline.

This study estimates excess mortality in Norway in 2024, using only pre-pandemic years as a reference. This provides a basis for identifying excess mortality related to COVID-19 and, consequently, informing Norway’s current COVID-19 strategy.

## Methods

Deaths per year were obtained from Statistics Norway’s table 10325 and grouped into age categories: 0, 1–19, 20–39, 40–64, 65–79, 80–89 and 90+ years, following NIPH's analysis framework [15].

We fitted a Bayesian negative binomial regression model to 2010–2019 mortality data, excluding 2011 for the 1–19 age group due to the 22 July terror attack. Deaths were modelled with a three-way interaction between year, age and sex, using a population offset and age-specific dispersion parameters. Minor modifications were made to the default priors from the R-package *brms* to improve convergence [17–19]. More details are available in Supplemental Material 1.

Expected mortality (per 100,000 people) was predicted for 2020–2024 using two approaches: (1) a conservative approach where the prediction for 2023 was carried forward to 2024, so that 2024 predictions assumed the 2010–2019 declining trend plateaued in 2023. (2) A non-conservative linear extrapolation to 2024.

## Results

**Figure 1.**
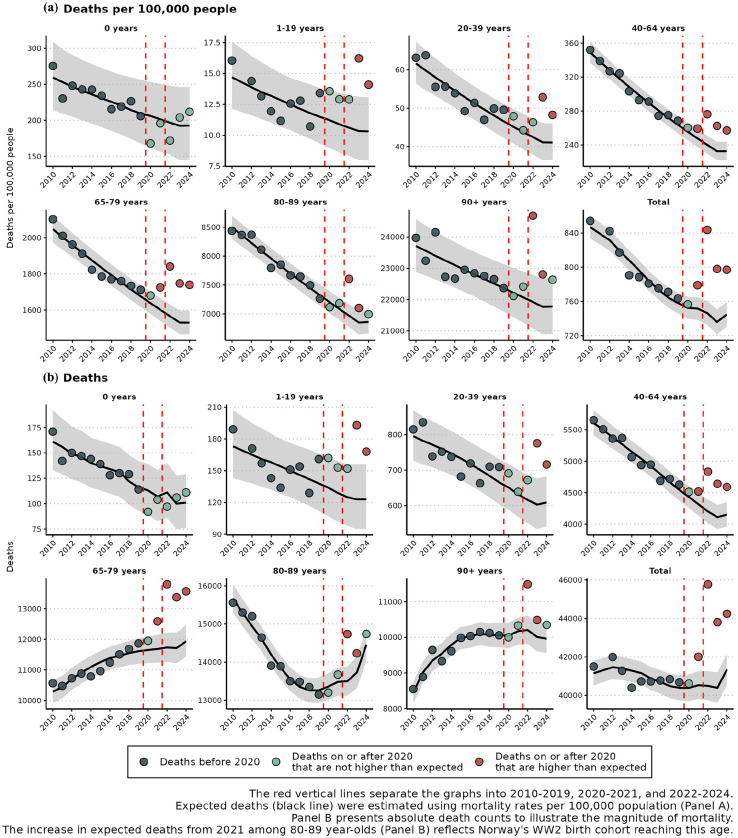
Observed and expected deaths in Norway by age group (a) per 100,000 and (b) total counts, with conservative expectations based on a 2010–2019 baseline extrapolated to 2023 and held constant thereafter.

Using the conservative approach, we estimated 2898 excess deaths in 2024 (7.0% excess; 95% prediction interval (PI), 4.9–9.1%). Significant excess mortality was observed in age groups 1–19 (45 deaths; 36.6% excess), 20–39 (107 deaths; 17.6% excess), 40–64 (439 deaths; 10.6% excess), and 65–79 (1,631 deaths; 13.7% excess) (Table I and Figure 1).

**Table I.** Observed and expected deaths in Norway in 2024, as well as absolute and relative deviation with 95% PIs, divided by age and sex. Expected mortality is calculated (A) conservatively based on a 2010–2019 baseline extrapolated to 2023 and held constant thereafter and (B) based on a 2010–2019 baseline extrapolated to 2024.

Age	Population	Deaths	Expected deaths		Deviation (number)		Deviation (%)	
			Est.	95% PI	Est.	95% PI	Est.	95% PI
**(A) Baseline linearly extrapolated to 2023 and then held (conservative approach)**								
**Total**								
0 years	52,408	111	101	76 to 129	10	−18 to 35	9.9	−14.0 to 46.1
1–19 years	1,191,874	168	123	95 to 156	45	12 to 73	36.6	7.7 to 76.8
20–39 years	1,483,617	716	609	541 to 683	107	33 to 175	17.6	4.8 to 32.3
40–64 years	1,786,136	4592	4153	3961 to 4350	439	242 to 631	10.6	5.6 to 15.9
65–79 years	779,683	13,565	11,934	11,430 to 12,453	1631	1112 to 2135	13.7	8.9 to 18.7
80–89 years	210,751	14,739	14,459	14,031 to 14,894	280	−155 to 708	1.9	−1.0 to 5.0
90+ years	45,734	10,351	9960	9557 to 10,376	391	−25 to 794	3.9	−0.2 to 8.3
Total	5,550,203	44,242	41,344	40,542 to 42,160	2898	2082 to 3700	7.0	4.9 to 9.1
**Male sex**								
0 years	26,771	66	53	36 to 74	13	−8 to 30	24.5	−10.8 to 83.3
1–19 years	612,858	97	79	57 to 106	18	−9 to 40	22.8	−8.5 to 70.2
20–39 years	758,644	504	429	371 to 492	75	12 to 133	17.5	2.4 to 35.8
40–64 years	909,889	2817	2503	2352 to 2659	314	158 to 465	12.5	5.9 to 19.8
65–79 years	381,650	7730	6678	6293 to 7081	1052	649 to 1437	15.8	9.2 to 22.8
80–89 years	91,498	7348	7257	6953 to 7570	91	−222 to 395	1.3	−2.9 to 5.7
90+ years	14,408	3625	3433	3221 to 3655	192	−30 to 404	5.6	−0.8 to 12.5
Total	2,795,718	22,187	20,437	19,878 to 21,017	1750	1170 to 2309	8.6	5.6 to 11.6
**Female sex**								
0 years	25,637	45	47	31 to 67	−2	−22 to 14	−4.3	−32.8 to 45.2
1–19 years	579,016	71	43	28 to 63	28	8 to 43	65.1	12.7 to 153.6
20–39 years	724,973	212	180	144 to 219	32	−7 to 68	17.8	−3.2 to 47.2
40–64 years	876,247	1775	1649	1533 to 1771	126	4 to 242	7.6	0.2 to 15.8
65–79 years	398,033	5835	5254	4936 to 5589	581	246 to 899	11.1	4.4 to 18.2
80–89 years	119,253	7391	7200	6902 to 7506	191	−115 to 489	2.7	−1.5 to 7.1
90+ years	31,326	6726	6525	6186 to 6879	201	−153 to 540	3.1	−2.2 to 8.7
Total	2,754,485	22,055	20,905	20,337 to 21,486	1150	569 to 1718	5.5	2.6 to 8.4
**(B) Baseline linearly extrapolated to 2024 (non-conservative approach)**								
**Total**								
0 years	52,408	111	99	73 to 128	12	−17 to 38	12.1	−13.3 to 52.1
1–19 years	1,191,874	168	120	91 to 154	48	14 to 77	40.0	9.1 to 84.6
20–39 years	1,483,617	716	590	520 to 666	126	50 to 196	21.4	7.5 to 37.7
40–64 years	1,786,136	4592	4025	3830 to 4226	567	366 to 762	14.1	8.7 to 19.9
65–79 years	779,683	13,565	11,668	11,149 to 12,204	1897	1361 to 2416	16.3	11.2 to 21.7
80–89 years	210,751	14,739	14,200	13,759 to 14,651	539	88 to 980	3.8	0.6 to 7.1
90+ years	45,734	10,351	9885	9461 to 10,322	466	29 to 890	4.7	0.3 to 9.4
Total	5,550,203	44,242	40,592	39,761 to 41,438	3650	2804 to 4481	9.0	6.8 to 11.3
**Male sex**								
0 years	26,771	66	52	34 to 73	14	−7 to 32	26.9	−9.6 to 94.1
1–19 years	612,858	97	78	55 to 106	19	−9 to 42	24.4	−8.5 to 76.4
20–39 years	758,644	504	415	356 to 480	89	24 to 148	21.4	5.0 to 41.6
40–64 years	909,889	2817	2423	2270 to 2582	394	235 to 547	16.3	9.1 to 24.1
65–79 years	381,650	7730	6488	6092 to 6903	1242	827 to 1,638	19.1	12.0 to 26.9
80–89 years	91,498	7348	7106	6793 to 7431	242	−83 to 555	3.4	−1.1 to 8.2
90+ years	14,408	3625	3397	3174 to 3629	228	−4 to 451	6.7	−0.1 to 14.2
Total	2,795,718	22,187	19,963	19,383 to 20,558	2,224	1629 to 2804	11.1	7.9 to 14.5
**Female sex**								
0 years	25,637	45	47	30 to 68	−2	−23 to 15	−4.3	−33.8 to 50.0
1–19 years	579,016	71	42	26 to 62	29	9 to 45	69.0	14.5 to 173.1
20–39 years	724,973	212	174	139 to 215	38	−3 to 73	21.8	−1.4 to 52.5
40–64 years	876,247	1775	1602	1482 to 1727	173	48 to 293	10.8	2.8 to 19.8
65–79 years	398,033	5835	5178	4845 to 5526	657	309 to 990	12.7	5.6 to 20.4
80–89 years	119,253	7391	7093	6783 to 7409	298	−18 to 608	4.2	−0.2 to 9.0
90+ years	31,326	6726	6487	6130 to 6860	239	−134 to 596	3.7	−2.0 to 9.7
Total	2,754,485	22,055	20,628	20,036 to 21235	1,427	820 to 2019	6.9	3.9 to 10.1

Est., estimated; PI, prediction interval.

**Figure 2.**
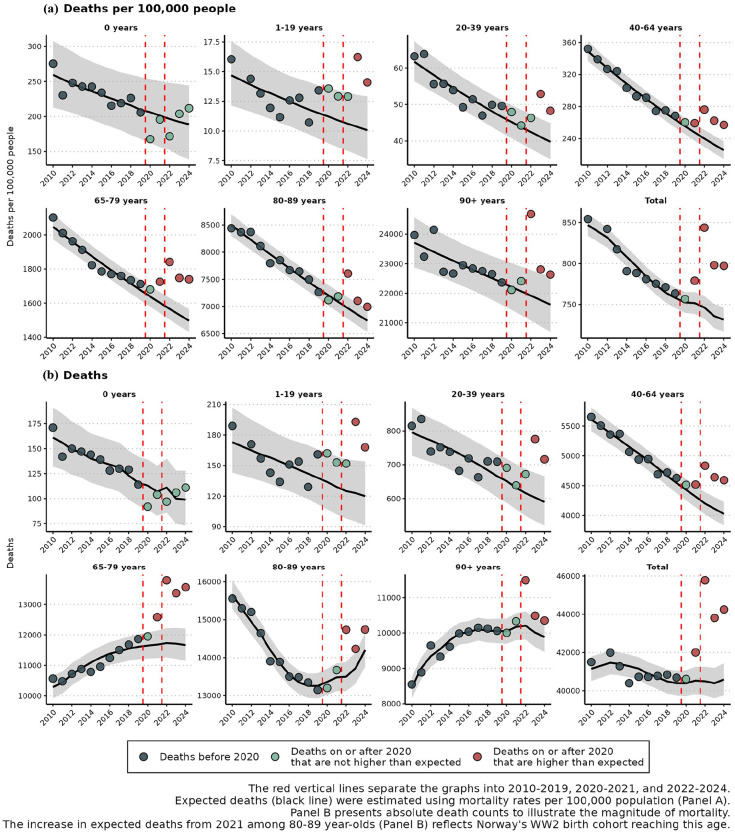
Observed and expected deaths in Norway by age group (a) per 100,000 and (b) total counts, with expectations based on a 2010–2019 baseline extrapolated to 2024.

With the non-conservative approach, we estimated 3650 excess deaths in 2024 (9.0% excess; 95% PI, 6.8–11.3%). Significant excess mortality was observed across age groups 1–19 (48 deaths; 40.0% excess), 20–39 (126 deaths; 21.4% excess), 40–64 (567 deaths; 14.1% excess), 65–79 (1,897 deaths; 16.3% excess), 80–89 (539 deaths; 3.8% excess) and 90+ (466 deaths; 4.7% excess) (Table I and Figure 2).

Under both approaches, ages 1–39 and 40–64 accounted for approximately 5% and 15% of total excess mortality, respectively (Table I).

Full sex- and age-specific results are available in Supplemental Material 1 (Figures S1–S4) and Supplemental Material 2.

## Discussion

The analysis demonstrates significant excess mortality in Norway during 2023–2024 across all age groups except infants. Whereas the elderly (65+) accounted for most excess deaths in absolute terms, the finding that approximately 20% of excess deaths occurred among those under 65 years is surprisingly high, representing a substantial burden in this younger population. The increase in excess mortality since SARS-CoV-2 became endemic in Norway in 2022 is especially concerning.

When comparing our estimates of excess mortality in Norway in 2023 with previous research in this field, our findings fell between existing published values. Specifically, our estimates were higher than the estimates reported by Knudsen et al. [13], but lower than the estimates from Strøm et al. [14], with detailed numerical comparisons provided in Table S1 (Supplemental Material 1).

Our findings align with broader Nordic patterns of disrupted mortality decline across Denmark, Finland, and Sweden since the post-acute pandemic phase, with all countries showing downward shifts in life expectancy—changes that are unusual from a historical perspective [20]. This suggests common regional drivers of persistent excess mortality rather than Norway-specific factors.

Although multiple factors may contribute to excess mortality, our 2010–2019 baseline period already captured the impact of seasonal infectious diseases, including influenza. Given the ‘return to normal’ conditions from 2022 onwards, including resumption of typical seasonal disease patterns, the key question is what has changed to cause sustained elevation above historical mortality levels? The persistent excess mortality observed from 2022–2024 cannot be explained by the return of pre-existing seasonal factors that were already present in our baseline period. The primary novel factor distinguishing the post-2022 period is SARS-CoV-2 and its potential long-term health consequences [21,22]. Pandemic-related effects from societal disruptions may also play a role. In Norway, significant excess sick leave in 2023 and primary healthcare consultations in 2024 have been observed in diagnoses associated with post-acute COVID-19 sequelae [23,24], typically increasing during or after COVID-19 waves, strengthening the COVID-19 hypothesis. However, without cause-specific mortality data, we cannot make definitive causal claims about the relative contributions of different factors.

NIPH’s approach to include 2023 as part of the reference basis for estimating excess mortality in 2024 does not account for the clear break in expected mortality trends in the years since 2022, blurring important changes from historical trends. This may overlook key developments. By using a baseline based solely on pre-pandemic years, our analysis provides a more accurate picture of mortality patterns after 2020.

The main limitation of this study is the use of aggregated cause-of-death data.

## Conclusion

Using a pre-pandemic reference period, significant excess mortality persisted across all ages except infants in Norway in 2024. The persistence of excess mortality from 2022–2024 indicates that a new elevated mortality baseline has been established, representing a reduction—or perhaps even reversal in some groups—of the long-standing mortality decline that characterised pre-pandemic Norway. Although multiple factors may contribute, given the sustained excess mortality since 2022, our findings suggest that the unmitigated spread of SARS-CoV-2 in Norway since 2022 can be associated with increased mortality, particularly for those under 65.

## Supplemental Material

sj-docx-1-sjp-10.1177_14034948251371830 – Supplemental material for Excess all-cause mortality in Norway in 2024 Supplemental material, sj-docx-1-sjp-10.1177_14034948251371830 for Excess all-cause mortality in Norway in 2024 by Richard Aubrey White, Anders B. Nygaard, Arne Søraas and Gunhild A. Nyborg in Scandinavian Journal of Public Health

sj-xlsx-2-sjp-10.1177_14034948251371830 – Supplemental material for Excess all-cause mortality in Norway in 2024 Supplemental material, sj-xlsx-2-sjp-10.1177_14034948251371830 for Excess all-cause mortality in Norway in 2024 by Richard Aubrey White, Anders B. Nygaard, Arne Søraas and Gunhild A. Nyborg in Scandinavian Journal of Public Health
