# Supplementary material for: Excess all-cause mortality in Norway in 2024
Source: Scand J Public Health. 2025 Sep 8;54(4):418–24. doi: 10.1177/14034948251371830 (PMC13176487; doi:10.1177/14034948251371830)

# Bayesian negative binomial regression model

## Model specification

We used a Bayesian negative binomial regression model implemented in the brms package to estimate expected death counts based on 2010–2019 data. For the 1–19 age group, data from 2011 were excluded to avoid bias from the July 22 terror attack. The model simultaneously estimated the mean and dispersion (shape) parameters of the negative binomial distribution using a distributional regression framework.

### Mean model structure

The mean model incorporated a fully saturated three-way interaction design:

**deaths_n ~ year_for_modelling × age_category × sex + offset(log(population))**

- year_for_modelling was coded as years since 2010.
- age_category comprised seven groups: 0, 1–19, 20–39, 40–64, 65–79, 80–89, and 90+ years.
- sex included male and female.
- The offset term adjusts for varying population sizes across strata, allowing the model to estimate rates rather than raw counts.

### Dispersion model structure

The shape (dispersion) parameter was modeled as varying by age group:

**shape ~ (1|age_category)**

This structure was motivated by overdispersion diagnostics from age-specific Poisson models. Overdispersion ratios—calculated using both deviance-based (residual deviance / degrees of freedom) and variance-based (residual variance / mean fitted value) methods—ranged from 0.32 (underdispersion in infants) to 4.07 (overdispersion in the oldest age group). A standard negative binomial model with a constant shape parameter would not adequately capture this heterogeneity.

## Prior specification and justification

Default priors from *brms* were used, with modifications to the dispersion model to improve convergence. They are weakly informative, chosen to improve convergence and sampling efficiency:

- Mean model: Regression coefficients received flat (improper uniform) priors. The intercept used a Student-t(3, -4.3, 2.5) prior, weakly informative and scaled to the observed distribution of the outcome.
- Dispersion model: Shape coefficients received flat priors. The shape intercept was given a Student-t(3, 0, 1) prior, and the random effect standard deviation used a Student-t(3, 0, 1) prior (both more regularizing than the default Student-t(3, 0, 2.5)) to improve model convergence. Results were substantively identical when using the default priors versus the modified priors.

**Table S1.** Comparisons of estimates of excess mortality in Norway in 2023 from White et al. 2025 against Strøm et al. 2024 and Knudsen et al. 2024

|  | **Strøm et al. 2024** | | **Knudsen et al. 2024** | | **White et al. 2025** | |
| --- | --- | --- | --- | --- | --- | --- |
| **Group** | **Number**  **(95% PI)** | **Relative excess**  **(95% PI)** | **Number**  **(95% PI)** | **Relative excess**  **(95% PI)** | **Number**  **(95% PI)** | **Relative excess**  **(95% PI)** |
| *Male* |  | 11.3%  (11.0% to 11.6%) | 1613  (648 to 2578) | 8.0%  (3.1% to 13.5%) | 2045  (1483 to 2594) | 10.3%  (7.3% to 13.5%) |
| *Female* |  | 7.1%  (6.8% to 7.5%) | 699  (-314 to 1713) | 3.3%  (-1.4% to 8.5%) | 1368  (789 to 1929) | 6.7%  (3.7% to 9.6%) |
| *0*  *years* |  |  |  |  | 6  (-22 to 31) | 6.0%  (-17.2% to 41.3%) |
| *1-19 years* |  |  |  |  | 70  (38 to 98) | 56.9%  (24.5% to 103.2%) |
| *0-19 years* |  |  | 37  (-87 to 162) | 14.6%  (-23.1% to 126.6%) |  |  |
| *20-39 years* |  |  | 68  (8 to 128) | 9.7%  (1.0% to 19.9%) | 173  (100 to 242) | 28.7%  (14.8% to 45.3%) |
| *1-39 years* | 252  (61 to 442) | 35.7%  (28.1% to 48.8%) |  |  |  |  |
| *40-64 years* |  |  | 186  (-89 to 460) | 4.2%  (-1.9% to 11.1%) | 528  (333 to 717) | 12.8%  (7.7% to 18.3%) |
| *65-79 years* |  |  | 1020  (692 to 1349) | 8.3%  (5.5% to 11.2%) | 1656  (1141 to 2155) | 14.1%  (9.3% to 19.2%) |
| *80-89 years* |  |  | 587  (192 to 981) | 4.3%  (1.4% to 7.4%) | 505  (92 to 912) | 3.7%  (0.7% to 6.8%) |
| *90+ years* |  |  | 77  (-438 to 591) | 0.7%  (-4.0 to 6.0%) | 476  (59 to 881) | 4.8%  (0.6% to 9.2%) |

**Figure S1.** Observed and expected deaths in Norway by age group within males: (A) per 100,000 and (B) total counts, with conservative expectations based on a 2010–2019 baseline extrapolated to 2023 and held constant thereafter.


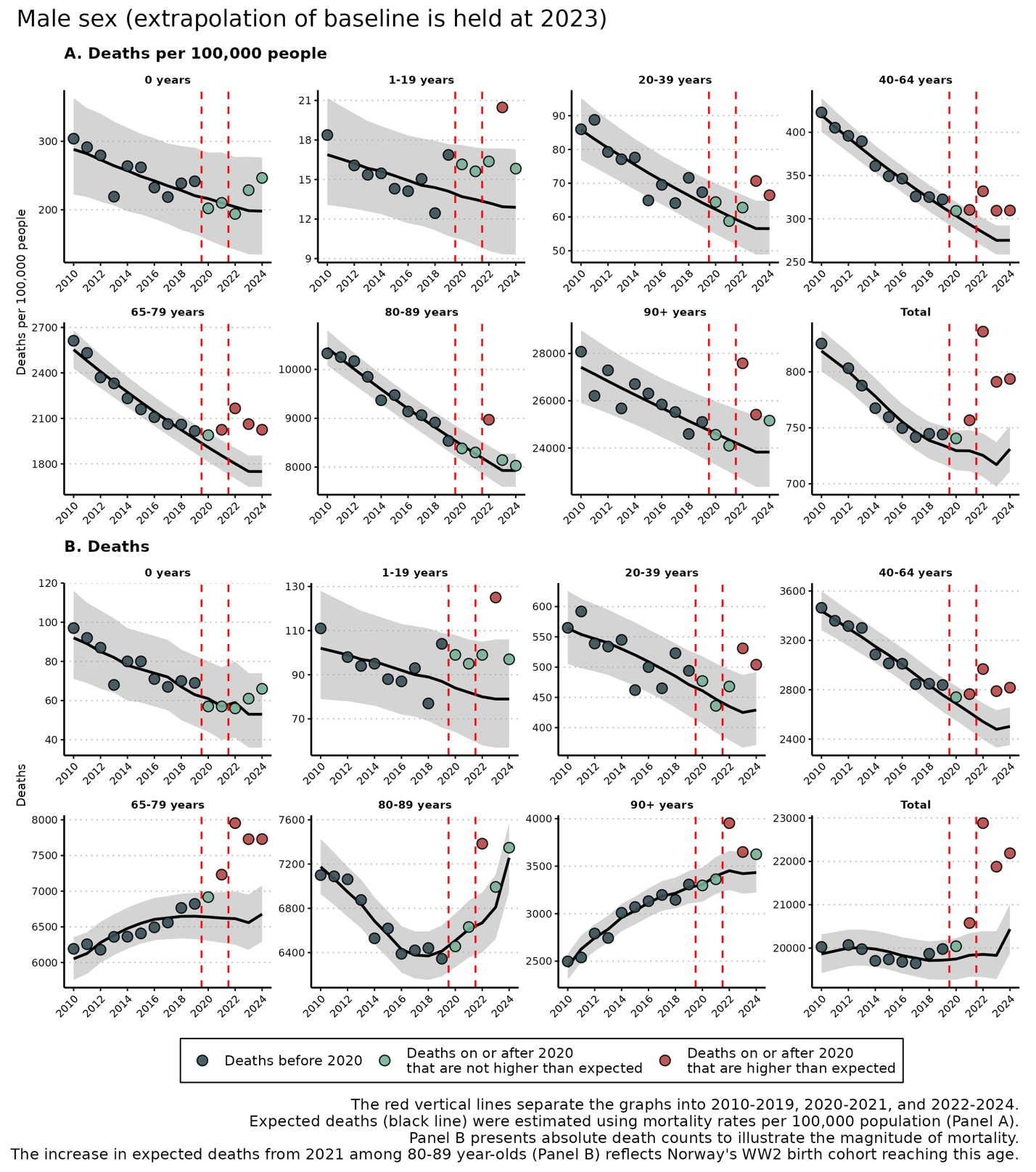


**Figure S2.** Observed and expected deaths in Norway by age group within males: (A) per 100,000 and (B) total counts, with conservative expectations based on a 2010–2019 baseline extrapolated to 2024.


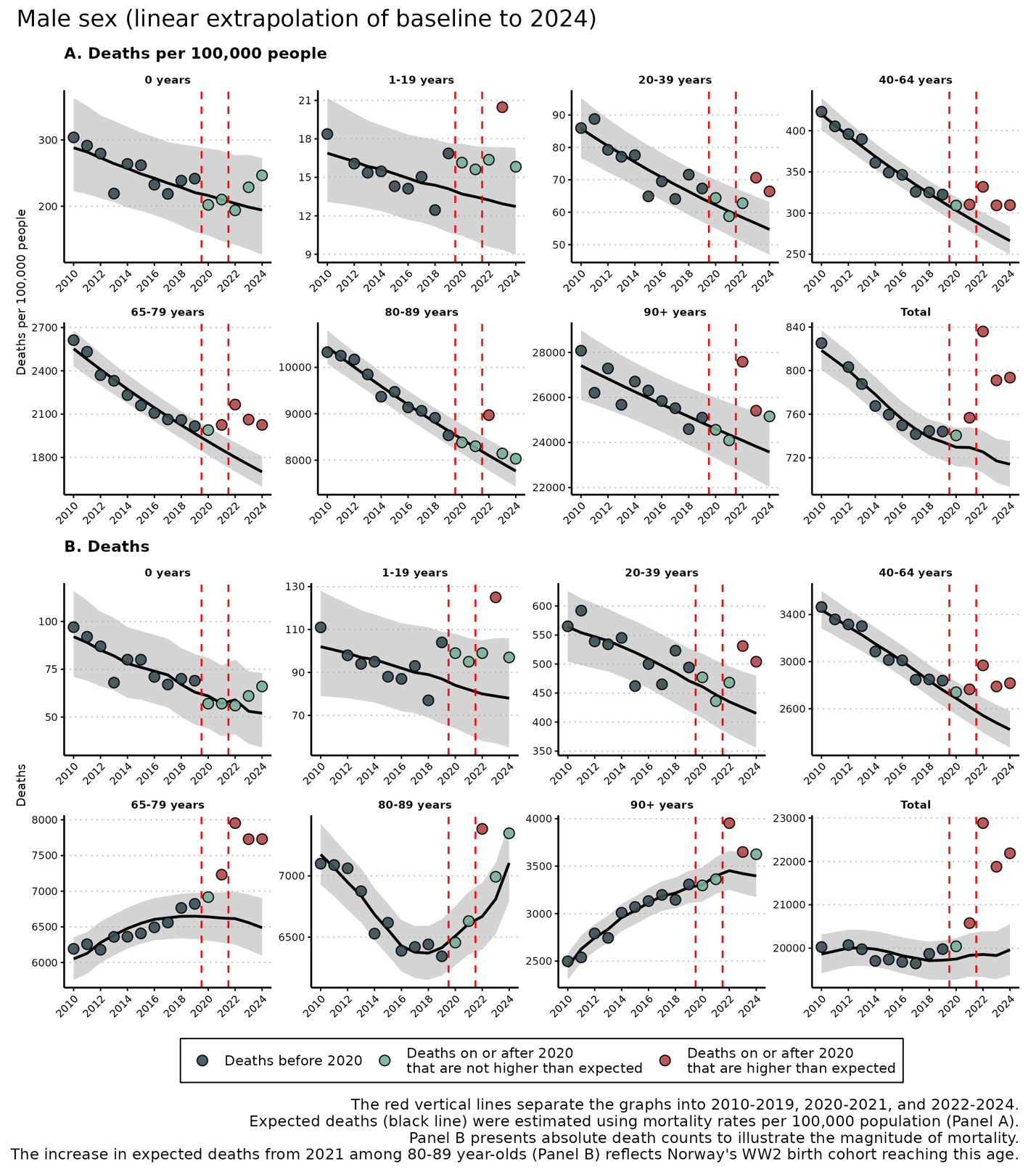


**Figure S3.** Observed and expected deaths in Norway by age group within females: (A) per 100,000 and (B) total counts, with conservative expectations based on a 2010–2019 baseline extrapolated to 2023 and held constant thereafter.


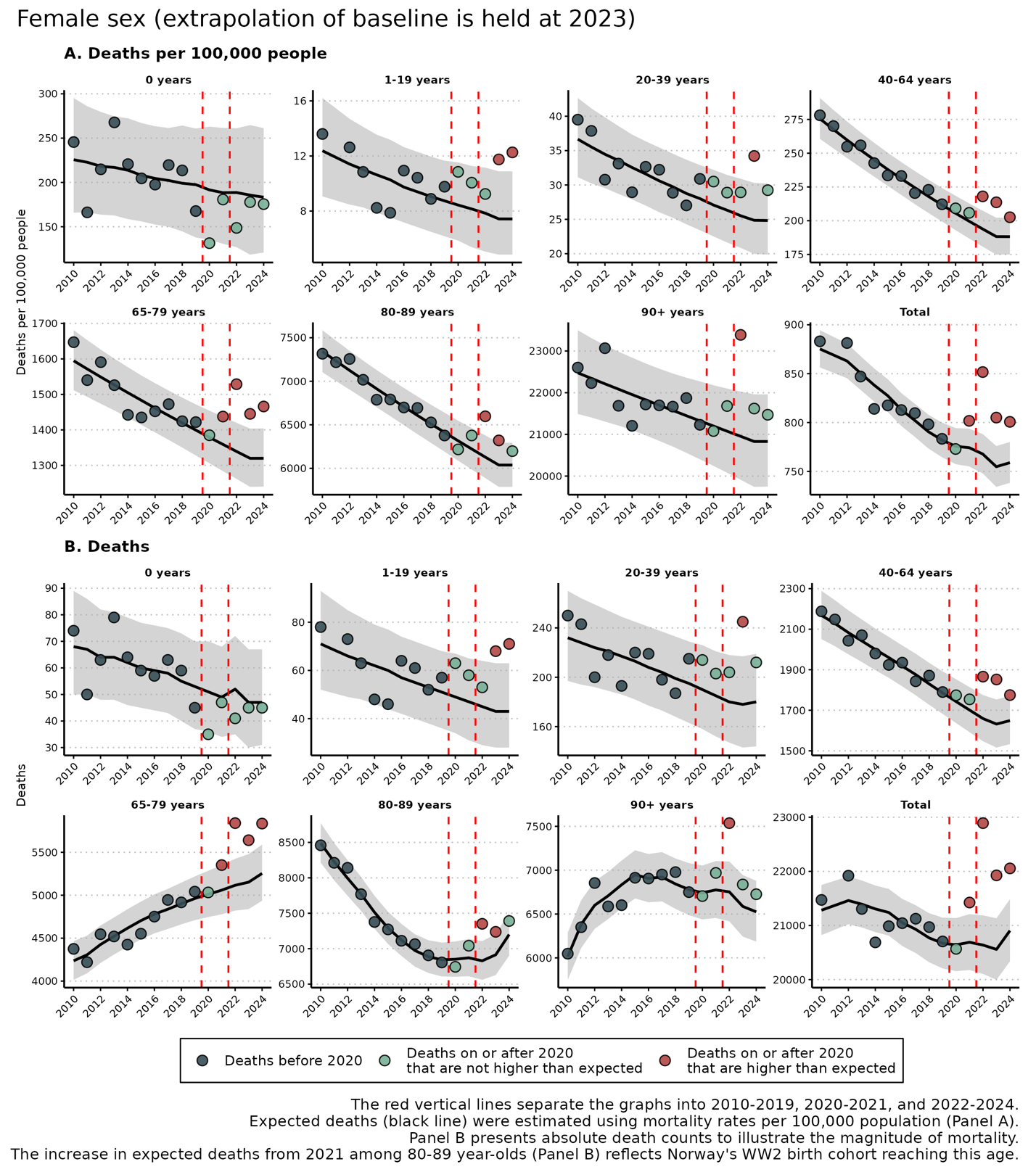


**Figure S4.** Observed and expected deaths in Norway by age group within females: (A) per 100,000 and (B) total counts, with conservative expectations based on a 2010–2019 baseline extrapolated to 2024.


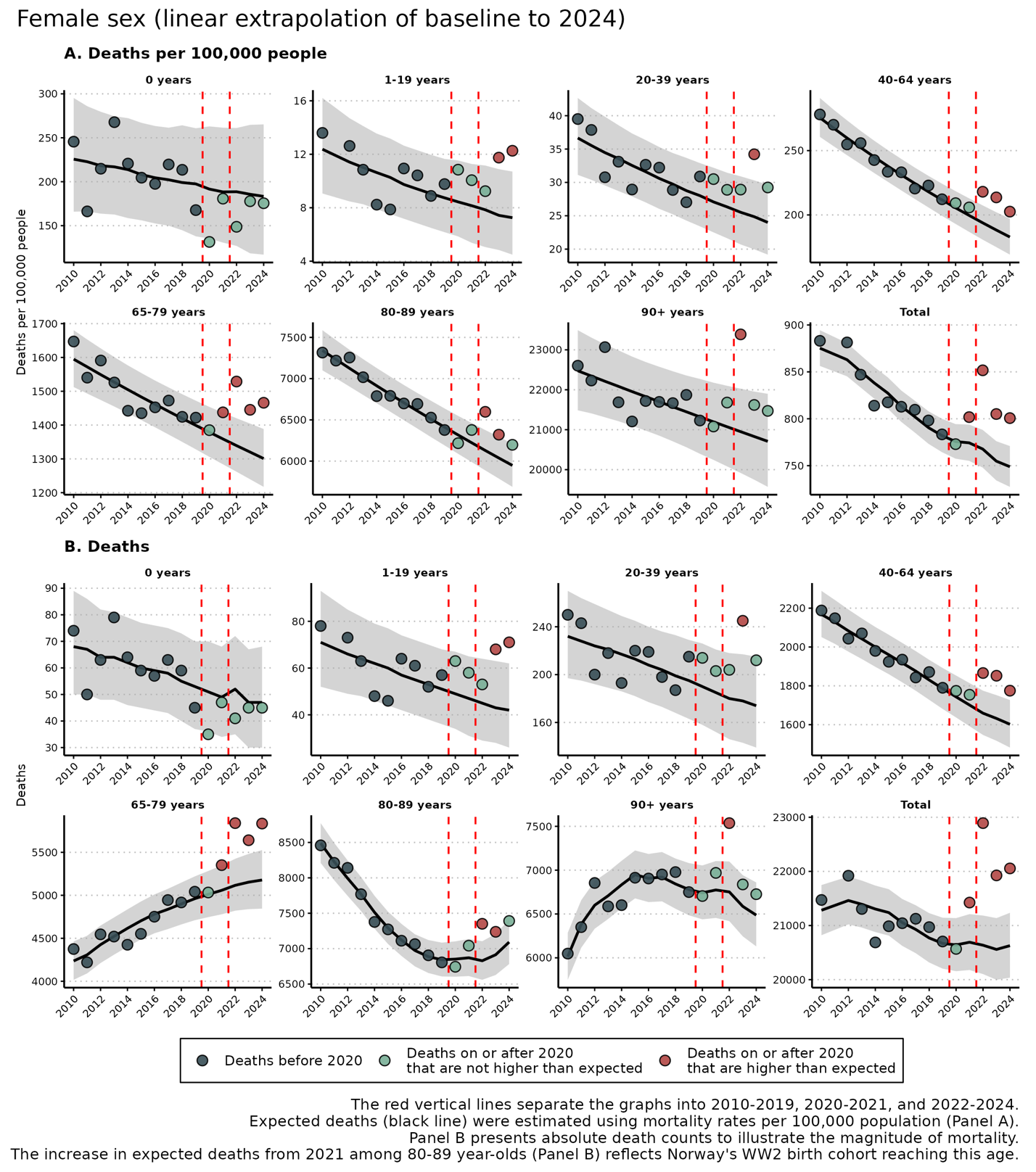

Supplement: sj-docx-1-sjp-10.1177_14034948251371830 – Supplemental material for Excess all-cause mortality in Norway in 2024 [file sj-docx-1-sjp-10.1177_14034948251371830.docx]
